# Ophthalmia Neonatorum

**Published:** 1872-02

**Authors:** 


### OPHTHALMIA NEONATORUM.

*A Clinical Lecture at the Dispensary of the Medical College of Ohio.*

BY PROFESSOR SEELY.

Gentlemen,—The first case I beg to call your attention to is this little child, three weeks old. I open the eye-lids, and you see from one eye the matter is streaming out; from the other, you might see it resting in the lower cul-de-sac.

We have before us a case of the so-called ophthalmia neonatorum.

What is the disease? From the physical characters, certainly all would say, a purulent inflammation of the conjunctiva.

I have already tried to impress on your minds some points in differential diagnosis in opthalmic troubles. In these little people, while the tendency of mucous inflammations is towards the purulent form, yet i t is by no means absolutely necessary for them all to assume it› for we sometimes find but a catarrhal form with only a muco-purulent discharge, and this may be slight. A simple statement of such a fact may be sufficient to afford some comfort when you are brought into contact with irritated eyes in your obstetric practice.

Let us see what the symptoms are which should attract your attention. If, in fro m twelve hours to a week or two after birth, you find the eye-lashes matted together, or the eyes tearing a good deal, and, on depressing the lower lids, the conjunctiva looks red and swollen, you have sufficient cause for suspecting the commencement of an inflammation. If these symptoms appear in the first three days, you would be warranted in regarding them as the beginning of what is most strictly termed ophthalmia neonatorum—purulent conjunctivitis in new-born children.

*Ætiology.*—Almost any irritation of so delicate a membrane may produce a purulent inflammation, and yet there are certain irritants to which the eyes of the new-born are especially liable. The child, in its passage through the vagina, comes in contact with whatever secretions may be there, and particles from these may be the exciting cause. These vaginal discharges are probably the most frequent cause of this disease, and the cases brought before you have only too frequently such an origin.

I have given the mother of the child a prescription for blenorrhagia, which she says she was suffering from when the child was born. So, in this case, we probably need look no further for a cause, as you all know the virulence of the gonorrhœal matter. But do not be too ready to infer such an origin, as carelessness in cleansing the child and caring for it, by exposing the eyes to too bright a light, allowing the cold air to strike, etc., may produce the character of inflammation. For your therapeutics, it matters little what the cause may be. If you have formed suspicions or not, you give the necessary precautions about the hands being clean when the child's eyes are attended to, and that all the cleansing apparatus be absolutely clean, and used only for the child. In this way, you protect those about, and also prevent reinoculation, if the mother has gonorrhæa. Gonorrhœal ophthalmia is only produced by some of the particles of matter coming in contact with the conjunctiva; and it is on this account that we caution, so strongly, patients with gonorrhœa against getting, in any way, the matter into the eyes.

*Prognosis.*—The prognosis will depend on the stage in which you see the case. Seen in the premonitory stage, it is always good, for the congestion can almost always be removed by very simple treatment; if not seen till the eye-lids are more or less thickened, and discharging pus, it is still good; but if the cornea shows any signs of being involved, the prognosis must be guarded.

*Treatment.*—In the first stage, a weak astringent—such as half a grain of zinc or nitrate of silver to the ounce of water, or even a weak solution of the sulphate of morphia (gr. ½ aq. ℥ i.)—will suffice to relieve all the difficulty, as a rule, if used every two or three hours.

If the second stage has come on, and there is more or less swelling of the lids and a purulent discharge, the indispensable condition is just what I insisted upon so strongly while talking to you at my Thursday's clinic, on discharges from the ear, *cleanliness.* Every few minutes the matter must be wiped away with a soft rag, and once every fifteen or twenty minutes the eye-lids should be gently opened, and some warm water allowed to run from the end of the rag over the ball, to wash the accumulated matter from the conjunctiva. This can all be done without waking the child.

*Local Applications.*—After you have thoroughly cleansed the eyes, in the milder forms, when the corneas are clear and the lids but little thickened, I recommend you to use—especially if you are not skillful in everting eyelids, or can only see your patient once or twice a week—a weak solution of nitrate of silver, gr. ½ or i. to ℥ i: a drop or two to be placed between the lids every half hour during the day if the solution is gr. ½, every hour or every two hours if it is gr. i. This plan succeeds well in a large number of cases, and can be carried out by the mother or nurse.

If there is a good deal of thickening of the conjunctiva, the papillæ prominent, your surest plan is to see the patient every day, place its head between your knees, as I do here by way of illustration, evert the lids—the upper and lower at the same time, or separately, as you are able; then brush them with a strong solution of the nitrate—a ten or fifteen grain solution of the pure, or a twenty or thirty of the mitigated—washing the solution off immediately with salt and water, and finally with pure water. Such an application as this once a day is sufficient—using in the meantime a collyrium of atropine or morphine (gr. ½ aq. ℥ i), two or three drops of which are to be gotten thoroughly *into* the eye three times a day. I have never seen any cases where the diptheritic element was insufficient to modify the plans of treatment marked out, necessitating the care insisted upon in purulent ophthalmia of adults.

Pure diphtheritic inflammation is never met with in newborn infants, but some of its characters are: For example, we sometimes find the lids pretty hard and hot, with a palish conjunctiva secreting a thin discharge of a filmy character, that quickly covers the surface of the membrane. Weaker, in such cases, recommends internally a small quantity of calomel three times a day, and the rubbing on the brow of a little mercurial ointment, discontinuing this treatment immediately the purulent discharge thoroughly begins. (See vol. 1, part 1, 2d edition Maladies des Yeux.) The all-important question in this is how to act when there is an affection of the cornea. Your attention must be continually directed to the state of the membrane, for in complications on its part lie the dangers. Here we want to get rid of the cause of the corneal affection—viz., the swelled lids and chemosed bulbous conjunctiva—as rapidly as possible; hence, we continue the applications and soothing wash, making use of extreme care if the lids are everted.

If there is an ulcer, a paracentesis of the anterior chamber should be made to relieve the intra-ocular tension.

If a perforation has occurred, and a prolapse of the iris exists, it should be snipped off.

The perforation may be large and the eye become staphylomatous, and require amputation.

The narration of a case that recently presented itself at my private rooms will be sufficient to put you on your guard in the after-treatment of eyes left with a corneal cicatrix.

A girl of thirteen had, while a child, some attacks of circumscribed keratitis, from which resulted rather a prominent corneal cicatrix. No special attention was paid to the eye, until the parents noticed an increase in its size. The cornea and pericorneal tissue had yielded, the anterior chamber had increased in size, and when I saw the case, the papilla was cupped and the eye was blind from glaucoma.

I have put you on your guard what are the objective symptoms to be looked for, as, in little children, no subjective ones are to be expected. Every eye that presents a corneal cicatrix, and especially an adherent one, the so-called leucoma adherens, should be looked after. Now, if in such an eye you at any time detect an increase in the intra-ocular tension, an iridectomy should be insisted upon at once. We had long been aware of the danger in such cases, but it remained for Grâefe the great teacher whom all mourn, to put in a clear light the exact relation between cause and effect, and to show the steps that lead from one to the other.—*The Clinic*, *February*, 1872.

